# Signal recognition particle prevents N-terminal processing of bacterial membrane proteins

**DOI:** 10.1038/ncomms15562

**Published:** 2017-05-18

**Authors:** Amitabh Ranjan, Evan Mercier, Arshiya Bhatt, Wolfgang Wintermeyer

**Affiliations:** 1 Department of Physical Biochemistry, Max Planck Institute for Biophysical Chemistry, 37077 Göttingen, Germany

## Abstract

Bacterial proteins are synthesized with an N-formylated amino-terminal methionine, and N-formylated peptides elicit innate-immunity responses against bacterial infections. However, the source of these formylated peptides is not clear, as most bacterial proteins are co-translationally deformylated by peptide deformylase. Here we develop a deformylation assay with translating ribosomes as substrates, to show that the binding of the signal recognition particle (SRP) to signal sequences in nascent proteins on the ribosome prevents deformylation, whereas deformylation of nascent proteins without signal sequence is not affected. Deformylation and its inhibition by SRP are not influenced by trigger factor, a chaperone that interacts with nascent chains on the ribosome. We propose that bacterial inner-membrane proteins, in particular those with N-out topology, can retain their N-terminal formyl group during cotranslational membrane insertion and supply formylated peptides during bacterial infections.

## Abstract (web summary)

Bacterial proteins are synthesized with formyl-methionine in their N-terminus, but most of them are co-translationally deformylated. Here, the authors show that inner-membrane proteins are protected from deformylation by the binding of the signal recognition particle (SRP) during translation.

The synthesis of all bacterial proteins is initiated with N-formyl-methionine (fMet), which during translation initiation is brought to the ribosome in the form of fMet-tRNA^fMet^. The formylated methionine at the N-terminus of nascent bacterial proteins is removed co-translationally shortly after the nascent protein has emerged from the peptide exit tunnel of the ribosome. Deformylation of nascent proteins is carried out by peptide deformylase (PDF), which binds to ribosome-nascent-chain complexes (RNCs) near the peptide exit. PDF is an essential enzyme in bacteria and, therefore, a promising target for antibacterial and anti-cancer drugs^1^. Deformylation, which pertains to around 95% of all bacterial proteins, in the majority of cases is followed by methionine excision by methionine aminopeptidase (MAP). As a result, >50% of all bacterial proteins are subject to N-terminal methionine excision^2^. The functional reason of using fMet-tRNA for translation initiation, rather than Met-tRNA, which is used for initiation in the eukaryotic cytosol and in archaea, is not clear. A recent, attractive hypothesis posits that the failure to remove the N-terminal fMet on nascent proteins constitutes the signal for degradation of such potentially misfolded proteins by an as yet unidentified downstream degradation pathway (‘fMet-degron' hypothesis^3^).

The nascent peptide chain emerging from the ribosomal peptide exit tunnel is also met by other ribosome-associated protein biogenesis factors (RPBs), such as the chaperone trigger factor or the signal recognition particle (SRP)^1^,^2^,^4^. RPBs bind to the ribosome in the vicinity of the tunnel exit, and it has been shown previously that PDF and MAP strongly compete for binding^5^, whereas trigger factor and SRP bind concurrently in an anti-cooperative fashion, weakening each otheŕs binding about ten-fold^5^,^6^. In contrast, concurrent binding of PDF with either trigger factor or SRP was observed on RNCs with practically no anti-cooperative effect^6^. While these results indicated that PDF binding to RNCs was not disturbed by the binding of SRP, the question remained whether there is competition for the nascent peptide which may interfere with deformylation. To examine this possibility, we have developed a deformylation assay using translating ribosomes as substrates and have compared the deformylation of nascent peptides on various RNCs differing in sequence and length of the nascent chain. We find a strong dependence of deformylation on both secondary structure and length of the nascent peptide. Most importantly, we observe that the deformylation of nascent peptides comprising SRP-specific signal sequences is virtually completely inhibited when SRP is bound to the nascent chain, while the deformylation of nascent peptides of other sequences is hardly affected. Trigger factor has no effect on deformylation and the inhibition by SRP. Our results suggest how bacterial proteins that are co-translationally inserted into the plasma membrane via the SRP pathway, in particular those with N-out topology, may escape deformylation, forming the source of formylated peptides during bacterial infections.

## Results

### Deformylation assay with RNC substrates

For examining PDF action on translating ribosomes, we have developed an assay for monitoring deformylation by PDF of N-formylated nascent peptides, based on radioactively labelled RNCs carrying f[^35^S]Met at the N terminus of the nascent peptide (Methods section). The extent of deformylation was measured by quantifying [^35^S]Met and f[^35^S]Met on TLC plates following proteinase K (PK) digestion of the nascent peptide (Methods section; Supplementary Fig. 1).

**Figure 1:**
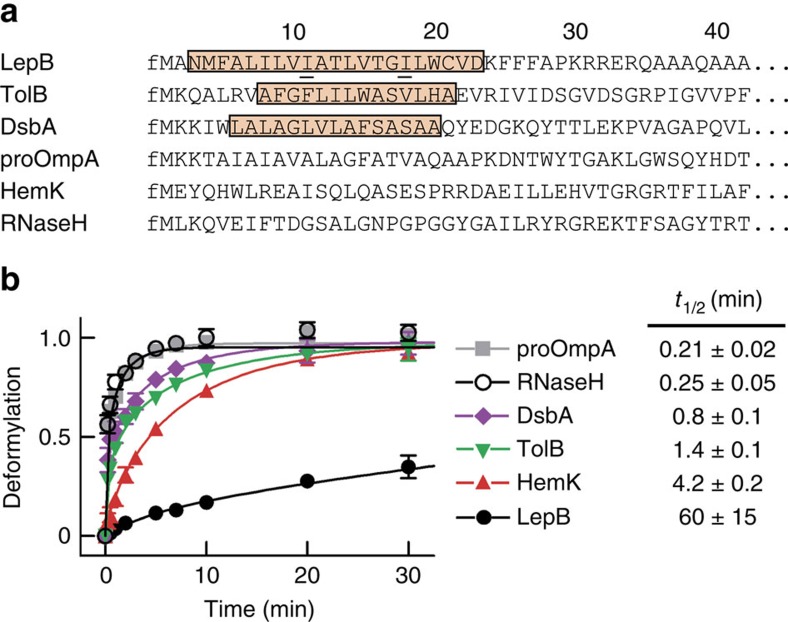
Deformylation of various RNCs with nascent peptides of 75 amino acids. (**a**) N-terminal sequences of RNCs. SRP-specific signal (TolB, DsbA) or signal-anchor (LepB) sequences are highlighted and the N-terminal formyl group is denoted by f. Positions where proline residues were inserted are underlined in the LepB sequence. (**b**) Time courses and *t*_1/2_ values of RNC deformylation. Assays were performed with 50 nM RNC carrying f[^35^S]Met at the N terminus and 10 nM PDF, and were analysed by TLC (Methods section; Supplementary Fig. 1). The extent of deformylation is normalized by the maximum extent of deformylation of the respective RNC after 60 min set to 1; in the case of LepB, the maximum deformylation was estimated by fitting the time course measured up to 150 min. For un-normalized time courses, Supplementary Fig. 2. Evaluation of the time courses (left panel), as described in Methods section, yielded *t*_1/2_ values (right panel). Error margins represent s.e.m. (*n*=2).

We have compared the deformylation of various RNCs exposing nascent *Escherichia coli* proteins differing in sequence and length. First we monitored time courses of deformylation for a number of RNCs carrying nascent peptides 75 amino acids in length. Among the RNCs tested, TolB and disulfide oxidoreductase A (DsbA) possess N-terminal SRP-specific signal sequences that are hydrophobic and tend to assume helical structures. Leader peptidase (LepB) is a type III inner-membrane protein with an SRP-specific signal-anchor sequence near the N terminus which, upon membrane insertion, assumes an N-out orientation. We have also tested RNCs that lack SRP-specific signal sequences: proOmpA, which carries an unstructured SecA-specific signal sequence at the N terminus, as well as RNaseH and HemK, both of which have unstructured N-terminal sequences that do not contain a signal sequence (Fig. 1a). To compare the deformylation of different RNCs on a reasonable timescale we used a low concentration of PDF, 10 nM, and a higher concentration of RNCs, 50 nM, for the assays and present the turnover rates as half-life times, *t*_1/2_ (Fig. 1b). These figures do not represent *in vivo* rates, but allow for comparisons of different RNCs, which show large variations, differing by about 20-fold (proOmpA versus HemK) or even 300-fold (proOmpA versus LepB). Previous studies on PDF using small non-ribosome-associated peptides as substrates have revealed that PDF prefers substrates with the sequence fMXZ, where X is any amino acid except D or E, and Z is K or R^7^,^8^. Our three most reactive RNCs (DsbA, proOmpA and RNaseH) meet these criteria, indicating that, at least qualitatively, RNC substrates recapitulate the preferences observed with model peptides. The next fastest RNC (TolB) lacks a favourable basic amino acid at position three of the nascent peptide, followed by HemK which has a disfavoured acidic amino acid in position two.

### Nascent peptide structure influences deformylation

**Figure 2:**
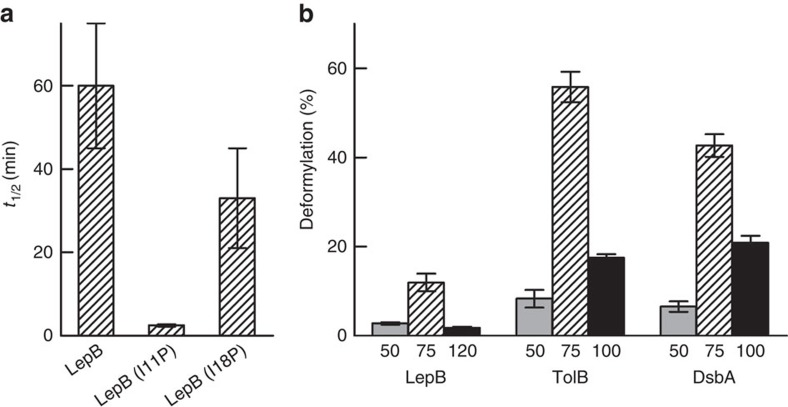
Deformylation of RNC variants. (**a**) Influence of proline substitutions in LepB75-RNC. Time courses of deformylation of LepB-, LepB(I11P)- and LepB(I18P)-RNC were measured and analysed as in Fig. 1b, and values of *t*_1/2_ are plotted. (**b**) Dependence of deformylation on the length of the nascent peptide. PDF assays with RNCs carrying nascent peptides of 50, 75 or 100/120 amino acids were performed as in Fig. 1b. The extent of deformylation after 30 min incubation with PDF is plotted. Error margins represent s.e.m. (*n*=2).

The use of RNC substrates of PDF also allowed for examining the influence of structural properties of nascent peptides on deformylation. The LepB75-RNC was found to be deformylated much more slowly than any of the other constructs, although the amino acid composition of the LepB peptide is not expected to be disfavoured by PDF^7^,^8^. Thus, the particularly low activity of the LepB75-RNC is presumably due to secondary structure (for example, α helix) formation near the N terminus that restricts the mobility of the nascent chain and impairs the access of the N-formylated Met residue to the active site of ribosome-bound PDF. In keeping with this, replacing Ile11 with Pro^9^ resulted in an increased rate (lowered *t*_1/2_) of deformylation (Fig. 2a), presumably by interrupting the ordered helical structure around the amino acid at position 11 and changing the orientation of the N terminus of the nascent peptide such that it can reach PDF more efficiently. An Ile-to-Pro substitution at position 18, two α-helical turns further down, had only a small effect, deformylation remaining slow (Fig. 2a). This indicates that the influence of secondary structure formation is stronger near the N terminus.

### Deformylation depends on nascent peptide length

To examine the effect of nascent-chain length on deformylation we used RNCs carrying shorter (50 amino acids) or longer (≥100 amino acids) nascent peptides (Fig. 2b). Deformylation was slow on RNCs with either short or long nascent peptides, compared to the faster deformylation observed with nascent peptides of 75 amino acids, of which about 40 may be exposed outside the tunnel (assuming a fully extended conformation of the peptide; Fig. 1a). Apparently, a nascent chain 50 amino acids in length, of which at most 15 are exposed outside the exit tunnel, is just sufficient but not yet optimal for the N terminus to reach the active site of ribosome-bound PDF. This is in keeping with early biochemical data which suggested that deformylation starts when the nascent chain reaches a length of 40–60 residues^10^,^11^. Furthermore, a structural model of the ribosome-PDF complex predicts that 13–15 amino acids suffice to bridge the distance from the tunnel exit to the active site of PDF^12^. The low activity observed with RNCs carrying nascent chains of ≥100 amino acids may indicate that in these RNCs the N-terminal fMet is shielded by (partial) folding of the nascent chain. Alternatively, sampling the larger space available for the longer nascent chains may take more time, slowing down the access of the N terminus to the active site of PDF.

### SRP prevents deformylation of nascent signal peptides

**Figure 3:**
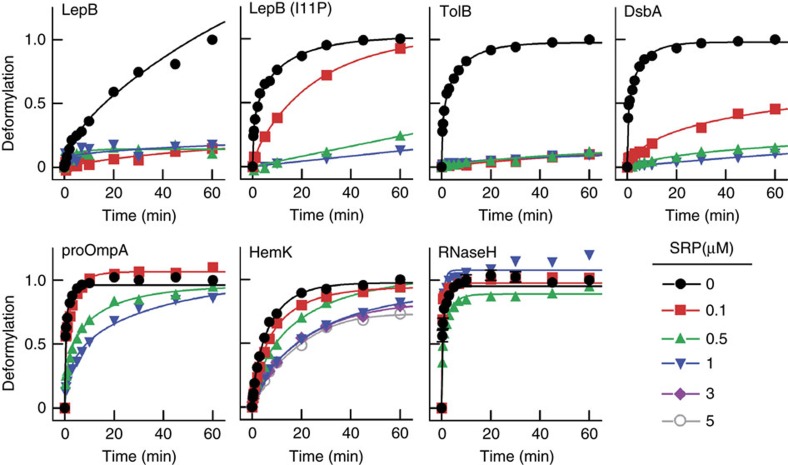
Effect of SRP on the deformylation of various RNCs. RNCs carrying nascent peptides of 75 amino acids were incubated with PDF in the presence of increasing amounts of SRP. The extent of deformylation over time is normalized to the maximum extent of deformylation after 60 min incubation with PDF and no SRP.

Next we examined the effect of SRP binding on deformylation by PDF, using RNC substrates with nascent peptides 75 amino acids in length. Increasing amounts of SRP were added to the RNCs and deformylation determined as above; as an example, the raw data obtained for the TolB-RNC are shown (Supplementary Fig. 1c). There were clearly two groups of RNCs (Fig. 3). In the group exposing SRP-specific signal peptides (LepB, TolB, DsbA), the presence of SRP led to complete or very strong inhibition even at the lowest SRP concentration, 0.1 μM. Apparently strong binding of SRP to the exposed signal peptide, which leads to a rather stable complex (half-life 5–10 s)^13^, prevents deformylation. To test the specificity of the SRP-mediated inhibition we examined the effect caused by the Ile-to-Pro exchange at position 11 of the LepB75-RNC (Fig. 3), which weakens SRP binding considerably^9^. The observed alleviation of inhibition supports the notion that SRP binding to the N-terminal signal peptide blocks deformylation. Presumably, the interaction with SRP restricts the mobility of the nascent peptide such that its movement towards PDF and the access to the catalytic site is impaired or prevented altogether.

RNCs without SRP-specific signal peptides showed much less (proOmpA, HemK) or no (RNaseH) inhibition of deformylation at low SRP concentration (0.1 μM) and a maximum of five-fold inhibition at saturating concentrations (1 μM or higher) of SRP (Fig. 3). This moderate inhibition is attributed to anti-cooperative binding of PDF and SRP to those RNCs, as previously observed for the concurrent binding of PDF and SRP to non-translating ribosomes^6^. Given the low concentration of SRP in the cell (about 0.1 μM), the small effect on the deformylation of nascent chains without signal sequences is probably not relevant *in vivo*.

### The SRP effect is not influenced by trigger factor

**Figure 4:**
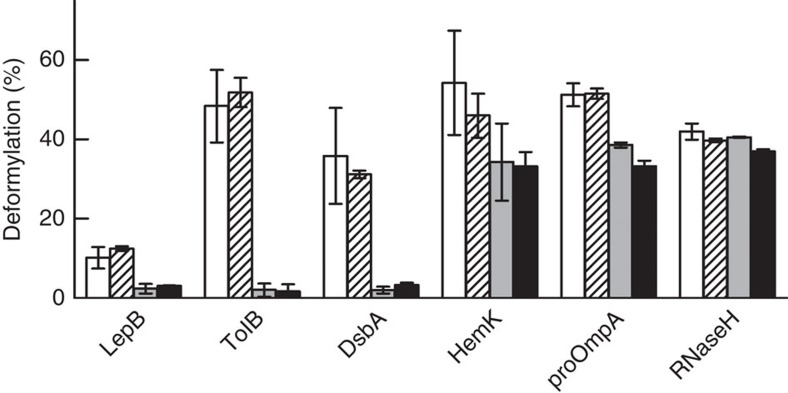
No effect of trigger factor on deformylation and its inhibition by SRP. RNCs carrying nascent peptides of 75 amino acids were reacted with PDF without any addition (open bars), or in the presence of trigger factor (hatched bars; 3 μM), SRP (grey bars; 1 μM) or both (black bars). The extent of deformylation after 30 min of incubation is plotted. Error bars represent s.e.m. (*n*=2).

The inhibitory effect of SRP was not influenced by the presence of trigger factor, even at high concentration (Fig. 4), independent of the nature of the nascent peptide. Furthermore, trigger factor alone did not affect deformylation by PDF, in line with previous observations^6^.

## Discussion

Previous studies have revealed that SRP and PDF can bind to LepB75-RNC concurrently with practically no effect on affinity^6^. Thus, the observed strong inhibition of deformylation by SRP on nascent chains containing signal or signal-anchor sequences cannot be due to ribosome-binding competition. Rather, the inhibition must result from SRP interacting with the nascent chain. The inhibition is observed at rather low SRP concentration, 0.1 μM, which is close to the *in vivo* concentration and indicates that the effect is relevant *in vivo*. We propose that during translation of SRP substrates in bacteria, early recruitment of SRP^9^,^14^ inhibits PDF-catalysed deformylation of the nascent chain, allowing N-terminally formylated proteins to be targeted to the translocon. Following membrane insertion, with or without inversion of transmembrane segments^15^, proteins with N-in topology may be accessible for PDF and deformylated. The N termini of membrane proteins with N-out topology, however, are not accessible to PDF and, therefore, more likely to remain N-formylated. An extracytosolic location of the formylated N termini also ensures that these proteins evade degradation by a cytosolic degradation system triggered by N-terminal fMet residues^3^,^16^.

While PDF is absent from the eukaryotic cytosol, there appears to be an analogous interplay between RPBs including SRP, MAP, the nascent polypeptide-associated complex and N-α-acyltransferases at the peptide exit of eukaryotic ribosomes^17^. Interestingly, methionine excision and subsequent acetylation has been shown to disrupt post-translational translocation across the ER in yeast, while SRP appears capable of protecting nascent chains from these modifications during cotranslational insertion into the ER membrane^18^. This latter point is consistent with the SRP-mediated inhibition of deformylation presented here and underscores the importance of signal peptide-independent early recruitment of SRP to bacterial ribosomes observed *in vitro* or to eukaryotic ribosomes shown *in vivo* by ribosome profiling^9^,^14^. Furthermore, our data show that deformylation is relatively ineffective with nascent peptides of a length of 50 amino acids. However, at this length of the nascent peptide SRP binds to RNCs synthesizing membrane proteins with high affinity^13^. This indicates that SRP can target these RNCs to the translocon for membrane insertion or translocation to the periplasm before deformylation by PDF becomes effective, despite rapid binding of PDF to the ribosome^5^.

Inner-membrane proteins make up about one quarter of the *E. coli* proteome, and a global topology analysis^19^ suggests that roughly 200 have N-out topology. Ribosome profiling of SRP-bound cargo provides a global overview of SRP substrates in *E. coli* and has recently identified 170 membrane proteins with N-out topology as SRP substrates^20^. Thus, SRP-mediated protection against deformylation and subsequent membrane insertion are likely to affect N-terminal modifications in a large number of *E. coli* proteins. We suggest that these proteins retain a formylated N terminus to variable extents. Among them the *E. coli* aspartate receptor^21^ (N-in topology) and LepB^22^ (N-out) were previously shown biochemically to be N-formylated. A recent proteomics investigation of N-terminal protein modification in *E. coli* revealed that retention of the N-terminal fMet is rare in general, being detected in only 54 of about 1,000 proteins under normal growth conditions^23^. Although the number of membrane proteins identified in that analysis was rather small (roughly 100), those with N-out topology were more likely to retain their fMet than N-in proteins.

The functional role in bacteria of retaining the formyl group on membrane or periplasmic proteins, if any, is not known. However, N-terminally unprocessed bacterial membrane proteins may serve as a source of short formylated peptides that are liberated by extracellular proteolysis and act as chemoattractants during bacterial infections^21^. Formyl peptides act by binding to formyl peptide receptors of leukocytes (neutrophils) and elicit chemotaxis, degranulation, reactive oxygen species production and phagocytosis^24^,^25^. Longer formylated signal peptides that are set free by signal peptidases and may escape deformylation due to their membrane location appear to serve similar functions^26^. Thus, the inhibition of deformylation by SRP binding to N-terminal signal sequences of nascent bacterial inner-membrane or periplasmic proteins may serve an important physiological role in innate-immunity responses directed against bacterial pathogens.

## Methods

### RNA constructs

The mRNAs and 4.5S RNA were transcribed *in vitro* from linear DNA templates and purified by ion exchange chromatography^27^. The DNA templates were PCR-amplified using the pUC19 plasmid (ThermoFisher Scientific) (for proOmpA, TolB, DsbA and 4.5S RNA) or the pET24a plasmid (Novagen) (for HemK, LepB and LepB variants) carrying the respective gene, using Phusion polymerase (NEB) together with a forward primer binding to the T7 promoter and a reverse primer placed according to the desired length of the mRNA^6^,^28^. The RNaseH construct was amplified from pET24a plasmid using analogous procedures.

### Expression and purification of proteins

PDF and Ffh were expressed in *E. coli* BL21 (DE3) pLysS strain (New England Biolabs) from pET24a plasmids^6^. PDF was purified through affinity chromatography using Talon affinity agarose (Clontech) and subsequent ion exchange chromatography on Q sepharose. CoCl_2_ (0.2 mM) was present throughout the purification^29^ which was necessary and sufficient to preserve the PDF activity. After purification, PDF protein was frozen in liquid nitrogen and stored at −80 °C in buffer A (25 mM HEPES, pH 7.5, 70 mM NH_4_Cl, 30 mM KCl, 7 mM MgCl_2_, 0.2 mM CoCl_2_ and 10% glycerol). Ffh was purified by Ni-NTA (Qiagen) affinity chromatography followed by ion exchange chromatography on SP sepharose^27^. Purified Ffh protein was frozen in liquid nitrogen and stored at −80 °C in buffer A without CoCl_2_. SRP was formed by adding a 1.2-fold excess of 4.5S RNA to Ffh protein in buffer A lacking CoCl_2_.

### Colorimetric PDF assay

The activity of purified PDF was assayed with a small formylated peptide substrate (fMLpNA), using a colorimetric assay based on the formation of p-nitroaniline according to published protocols^30^. In brief, PDF (10 nM) was incubated with fMLpNA (50–500 μM) in buffer A with 1 mM TCEP (Tris(2-carboxyethyl)phosphine). *Aeromonas* aminopeptidase was added (0.8 U ml^−1^) to convert the deformylated product (MLpNA) to p-nitroaniline. The absorbance of p-nitroaniline was monitored at 405 nm for 5 min at 37 °C. The rates obtained from different substrate concentration were plotted against the concentration of substrate and fitted to a hyperbolic equation to provide values for *K*_M_ (50 μM) and *k*_cat_ (5 s^−1^), which are comparable to published values^29^.

### RNC preparation

RNCs were prepared by *in vitro* translation using purified translation components from *E. coli*. All components (ribosomes; initiation factors IF1, IF2, IF3; elongation factors EF-Tu, EF-G; f[^35^S]Met-tRNA^fMet^; and total aminoacyl-tRNA containing ^14^C-labelled Leu-tRNA for quantification of charging efficiency) were prepared according to published procedures^13^. RNCs were prepared by *in vitro* translation of mRNAs lacking stop codons and purified by sucrose cushioning^9^. RNC concentrations were determined by quantification of ^35^S in liquid scintillation counting and relating it to the total concentration of ribosomes measured by absorbance. In the RNC preparations used in this work >60% of the ribosomes carried the specified nascent peptide.

### RNC deformylation assay

To monitor the enzymatic removal of the N-terminal formyl group of f[^35^S]Met-labelled nascent peptides by PDF we developed an assay using RNCs as substrates. RNCs (50 nM) were incubated with PDF (10 nM) at 37 °C in buffer A to which TCEP (1 mM) was added. The reaction was quenched by quickly boiling the samples at 99 °C, which rapidly quenched the reaction. The quenched samples were diluted by adding an equal volume of buffer A with 10 mM CaCl_2_ and 1 mM TCEP and digested with PK (20 μg μl^−1^) at 37 °C overnight. fMet was separated from Met by TLC (TLC silica gel 60, Merck) using n-butanol:acetic acid:water (3:1:1) as mobile phase (Supplementary Fig. 1a). With proOmpA-, TolB- and DsbA-RNC, a formylated peptide (fMet-peptide) was formed by PK treatment (see the example of TolB-RNC in Supplementary Fig. 1b), presumably due to a lysine residue next to the N-terminal fMet that inhibited the cleavage of the peptide bond between fMet and Lys. Apparently, deformylation by PDF rendered that bond PK-sensitive and the formation of Met was correlated with the disappearance of the (unlabelled) peptide (Supplementary Fig. 1b). For these cases, where fMet-peptide rather than fMet was produced by PK treatment, the total methionine was computed as (Met+fMet+fMet-peptide), rather than as (Met+fMet) as in the other cases.

A small amount of f[^35^S]Met-tRNA^fMet^ in initiation complexes that did not enter translation was present in the RNC preparations. To quantify this, the RNCs were treated with 2 mM puromycin for 30 minutes at 37 °C. The released f[^35^S]Met-puromycin was separated from the immobile f[^35^S]Met-nascent-chain-puromycin by TLC, allowing their quantification. The corrected value for f[^35^S]Met in nascent chains (max. correction 20%) was included in calculating the extent of deformylation. Oxidized fMet, which was present in the samples prior to, and remained constant during, PDF treatment was not included in the quantification (not shown in Supplementary Fig. 1). For the standards on TLC plates, f[^35^S]Met was generated by alkali treatment of f[^35^S]Met-tRNA^fMet^ and [^35^S]Met was available commercially (PerkinElmer).

### Analysis of time courses

Due to their complexity, multiple-turnover time courses cannot be fit by simple equations. Thus, deformylation time courses were analysed by numerical integration using Global Kinetic Explorer (KinTek). The minimal model required to fit all time courses was an extended Michaelis–Menten model including product release (E+S=ES→EP=E+P), involving five independent rate constants. Interpolation of each fitted curve provided a *t*_1/2_ value. While each time course is insufficient to define individual rate constants, the end level was reasonably constrained. The upper and lower boundaries of each end level were determined using the FitSpace Editor of the software and a normalized *χ*^2^ threshold of 1.2. The upper and lower boundaries of each end level were used to interpolate error margins for each *t*_1/2_ value.

### Data availability

The authors declare that all relevant data are available in the paper and its Supplementary Information files, or from the corresponding author upon request.

## Additional information

**How to cite this article:** Ranjan, A. *et al*. Signal recognition particle prevents N-terminal processing of bacterial membrane proteins. *Nat. Commun.* **8**, 15562 doi: 10.1038/ncomms15562 (2017).

**Publisher's note**: Springer Nature remains neutral with regard to jurisdictional claims in published maps and institutional affiliations.

## Supplementary Material

Supplementary Information Supplementary Figures
