# Supplementary material for: Signal recognition particle prevents N-terminal processing of bacterial membrane proteins
Source: Nat Commun. 2017 May 18;8:15562. doi: 10.1038/ncomms15562 (PMC5454389; doi:10.1038/ncomms15562)
Supplement: Supplementary Information — Supplementary Figures [file ncomms15562-s1.pdf]

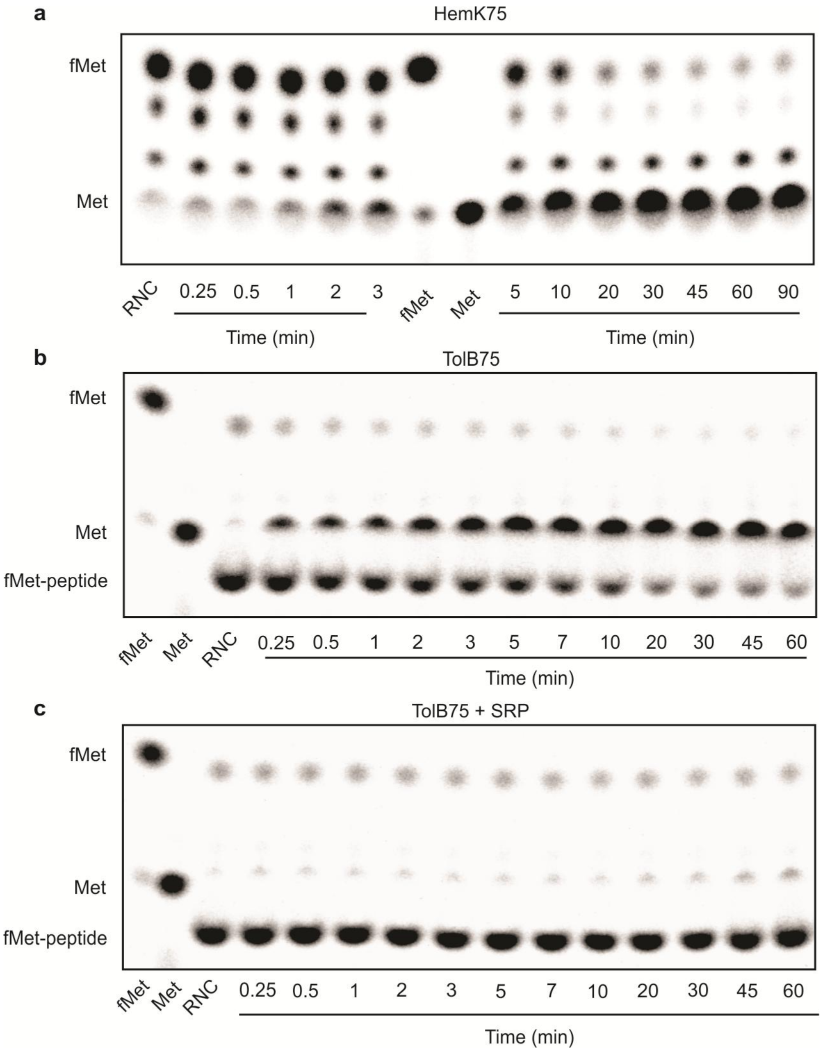

Supplementary Figure 1. Time courses of RNC deformylation as analyzed by thin-layer chromatography. (a) HemK75-RNC. The RNC was treated with PDF and digested with PK (Methods). fMet and Met were separated by TLC and quantified by phosphor imaging to determine f[^{35}S]Met and [^{35}S]Met using Multi-gauge software. Material migrating directly below fMet is assigned to residual formylated peptide that disappears upon deformylation and included in the quantification. The constant spot migrating directly above Met represents ^{14}C-labeled Leu which is included in the preparation of aminoacyl-tRNA to enable quality control; in panel (b) ^{14}C-labeled Leu is not seen because it runs in the spot of f[^{35}S]Met-peptide. RNC, sample digested with PK without prior PDF treatment. (b) TolB75-RNC. PK cleavage of the formylated nascent protein yielded fMet-peptide, and deformylation was indicated by the increasing amount of Met set free by PK. (c) Inhibition of deformylation of TolB75-RNC by SRP binding. fMet-peptide was formed by PK treatment, as in b, but was not deformylated during treatment with PDF in the presence of SRP.

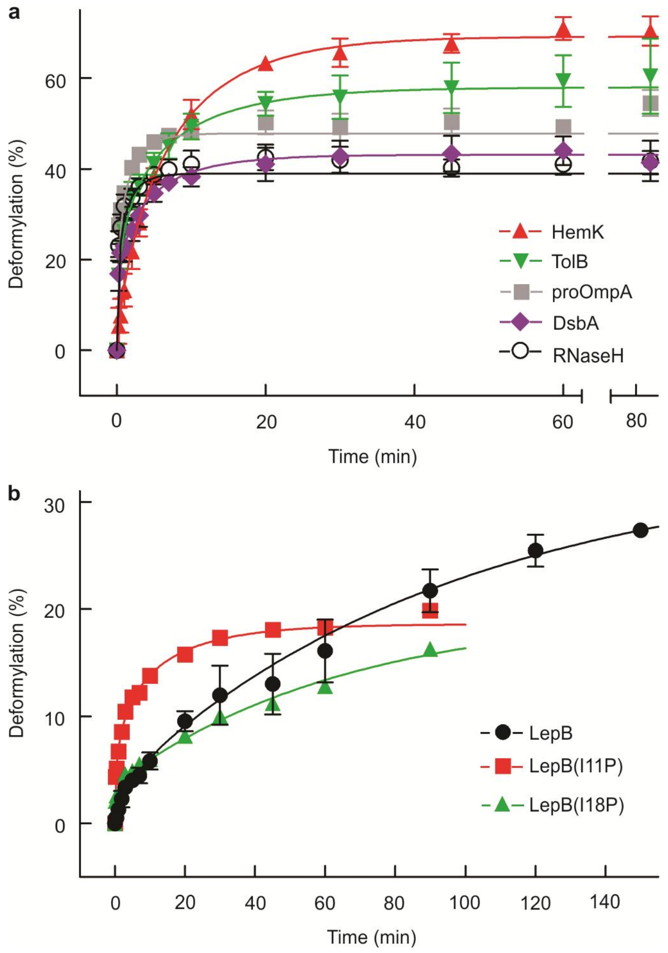

Supplementary Figure 2. Non-normalized deformylation time courses of various RNCs carrying nascent peptides of 75 amino acids. (a) TolB, DsbA, proOmpA, HemK, and RNaseH. (b) LepB, LepB(I11P), LepB(I18P). The ratio of Met/(Met + fMet) (LepB, HemK, RNaseH) or Met/(Met + fMet + fMet-peptide) (TolB, DsbA, proOmpA), determined as described above and corrected for methionine present at time zero (<5%), is plotted over time.
